# Diabetes, Metformin and the Clinical Course of Covid-19: Outcomes, Mechanisms and Suggestions on the Therapeutic Use of Metformin

**DOI:** 10.3389/fphar.2022.784459

**Published:** 2022-03-09

**Authors:** Clifford J. Bailey, Mike Gwilt

**Affiliations:** Life and Health Sciences, Aston University, Birmingham, United Kingdom

**Keywords:** type 2 diabetes, metformin, COVID—19, safety, SARS—CoV—2

## Abstract

**Objectives:** Pre-existing or new diabetes confers an adverse prognosis in people with Covid-19. We reviewed the clinical literature on clinical outcomes in metformin-treated subjects presenting with Covid-19.

**Methods:** Structured PubMed search: metformin AND [covid (ti) OR covid-19 (ti) OR covid19 (ti) OR coronavirus (ti) OR SARS-Cov2 (ti)], supplemented with another PubMed search: “diabetes AND [covid OR covid-19 OR covid19 OR coronavirus (i) OR SARS-Cov2 (ti)]” (limited to “Clinical Study”, “Clinical Trial”, “Controlled Clinical Trial”, “Meta-Analysis”, “Observational Study”, “Randomized Controlled Trial”, “Systematic Review”).

**Results:** The effects of metformin on the clinical course of Covid-19 were evaluated in retrospective analyses: most noted improved clinical outcomes amongst type 2 diabetes patients treated with metformin at the time of hospitalisation with Covid-19 infection. These outcomes include reduced admission into intensive care and reduced mortality in subgroups with versus without metformin treatment.

**Conclusion:** The pleiotropic actions of metformin associated with lower background cardiovascular risk may mediate some of these effects, for example reductions of insulin resistance, systemic inflammation and hypercoagulability. Modulation by metformin of the cell-surface ACE2 protein (a key binding target for SARS-CoV 2 spike protein) *via* the AMP kinase pathway may be involved. While pre-existing metformin treatment offers potentially beneficial effects and can be continued when Covid-19 infection is not severe, reports of increased acidosis and lactic acidosis in patients with more severe Covid-19 disease remind that metformin should be withdrawn in patients with hypoxaemia or acute renal disease. Prospective study of the clinical and metabolic effects of metformin in Covid-19 is warranted.

## Graphical Abstract

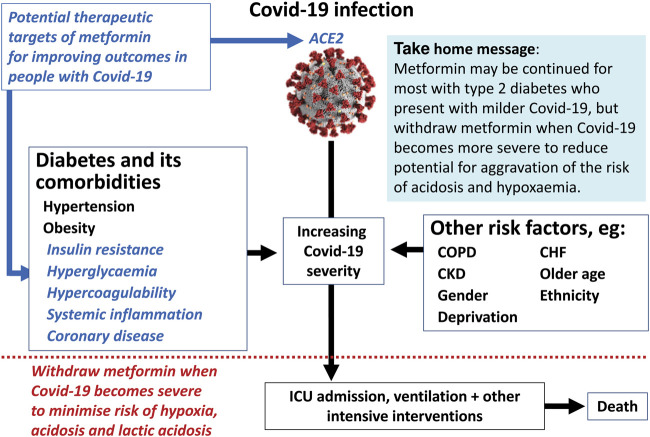

## Introduction

Covid-19 has infected millions of people world wide (Covid live, 2021), and it is well recognised that people with type 2 diabetes are more susceptible to Covid19 infection and worse outcomes (Apicella et al., 2020; Feldman et al., 2020). A growing evidence base of real-world studies has explored the effects of diabetes and metformin, the most commonly used treatment in type 2 diabetes, on the clinical course of Covid-19. We have reviewed the current literature on this topic and summarised the latest data on the impacts of diabetes and metformin on Covid-19 outcomes to derive pragmatic proposals for when to continue or withdraw treatment with metformin.

## Methods

This review is based on a structured literature search. Studies on the effects of metformin on outcomes in people infected with Covid-19 were identified by searching PubMed using the following search string: *metformin AND* (*covid [ti] OR covid-19 [ti] OR covid19 [ti] OR coronavirus [ti] OR SARS-Cov2 [ti]*), and *diabetes AND* (*covid OR covid-19 OR covid19 OR coronavirus [ti] OR SARS-Cov2 [ti]*); the second search was limited to “Clinical Study, Clinical Trial, Controlled Clinical Trial, Meta-Analysis, Observational Study, Randomized Controlled Trial, and Systematic Review” to exclude general reviews and limit the number of search hits. The 132 hits (April 14 2021) were examined for relevance. Reviews and systematic reviews were checked for additional references. Studies on the pleiotropic properties of metformin were from the reviews cited.

## Diabetes and Covid-19 Outcomes

### Prognosis in Patients With Covid-19 Complicated by Diabetes

**FIGURE 1:**
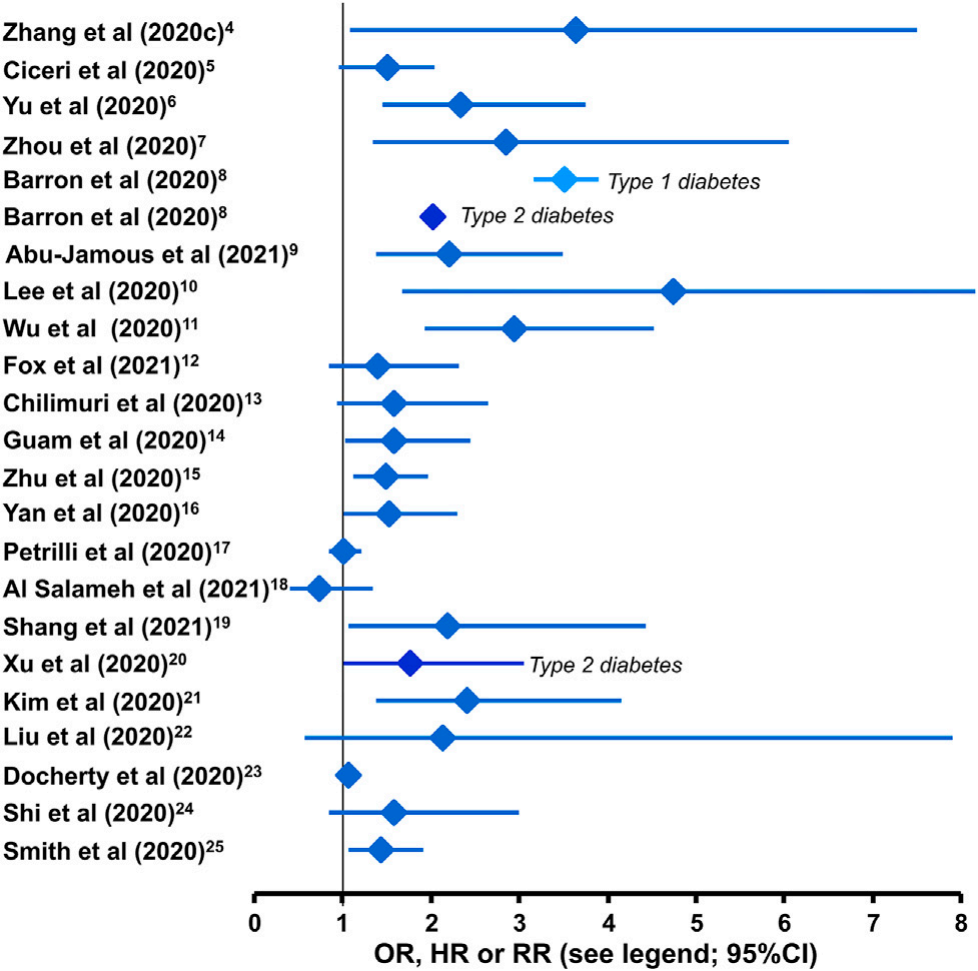
Impact of comorbid diabetes on the risk of mortality from Covid-19. Studies reporting morality outcomes [or composite of death, renal replacement therapy/haemodialysis, ventilation, intubation or use of vasopressor (Abu-Jamous et al., 2021)] are hazard ratios [HR (Zhang et al., 2020a; Barron et al., 2020; Ciceri et al., 2020; Yu et al., 2020; Zhou et al., 2020; Abu-Jamous et al., 2021)], odds ratios [OR (Guan W. J. et al., 2020; Wu J. et al., 2020; Chilimuri et al., 2020; Docherty et al., 2020; Kim et al., 2020; Lee et al., 2020; Liu et al., 2020; Petrilli et al., 2020; Shi et al., 2020; Xu et al., 2020; Yan et al., 2020; Zhu et al., 2020; Al-Salameh et al., 2021; Fox et al., 2021; Shang et al., 2021)], or relative risks [RR (Smith et al., 2020)] after stratification of populations for the presence or absence of diabetes are shown here. Diabetes was not stratified into types 1 and 2, except where stated.

Diabetes, among other comorbidities, increases the risk of a serious adverse prognosis for people who contract Covid-19, as shown by numerous individual studies, meta-analyses and systematic reviews (Figure 1). (Zhang et al., 2020a; Ciceri et al., 2020; Yu et al., 2020; Zhou et al., 2020; Barron et al., 2020; Abu-Jamous et al., 2021; Lee et al., 2020; Wu J. et al., 2020; Fox et al., 2021; Chilimuri et al., 2020; Guan W. J. et al., 2020; Zhu et al., 2020; Yan et al., 2020; Petrilli et al., 2020; Al-Salameh et al., 2021; Shang et al., 2021; Xu et al., 2020; Kim et al., 2020; Liu et al., 2020; Docherty et al., 2020; Shi et al., 2020; Smith et al., 2020; Saha et al., 2021; Liu et al., 2021; Kaminska et al., 2021; Elezkurtaj et al., 2021; Corona et al., 2021; Sun et al., 2021; Bud et al., 2020; Palaiodimos et al., 2020; Miller et al., 2020; Varikasuvu et al., 2021; Hussain et al., 2020; Shang et al., 2020; Guo L. et al., 2020; Aggarwal et al., 2020; Zaki et al., 2020; Parveen et al., 2020; Wu et al., 2021; Mantovani et al., 2020; Huang I. et al., 2020; Kumar et al., 2020; Roncon et al., 2020) Populations of patents hospitalised or entering intensive care for Covid-19 include a relatively high prevalence of diabetes, typically 1.5-3 fold greater than similar populations without diabetes (Carey et al., 2018; Abdi et al., 2020; Apicella et al., 2020; Bhatti et al., 2020; Wu C. et al., 2020; Hussain et al., 2020; Sathish and Cao, 2021; Sathish et al., 2021). Greater hyperglycaemia immediately prior to and during hospitalisation has consistently been associated with increased severity of Covid-19 infection in adults of any age with type 1 and type 2 diabetes, and especially in younger adults (<50 years of age) (Ando et al., 2021; Diedisheim et al., 2021; Gregory et al., 2021; Rawshani et al., 2021). The increased severity of Covid-19 infection in adults with diabetes has been noted irrespective of gender, ethnicity and geographical region (Ando et al., 2021; Diedisheim et al., 2021; Gregory et al., 2021; Rawshani et al., 2021).

In addition to escalating the hyperglycaemia in diabetes patients it is noted that newly-diagnosed diabetes is a common finding among people admitted for Covid-19, consistent with population-based surveys reporting a high prevalence of undiagnosed diabetes (Chou et al., 1994; Centers for Disease Control, 2020). The prevalence of diabetes is higher in older than younger patients with Covid-19, as would be expected, which has relevance for the clinical management of this population (Desai et al., 2020).

Figure 1 shows the risk of death from Covid-19 according to the presence versus absence of diabetes from retrospective studies. Overall, the risk of death from Covid-19 appeared to be at least doubled when diabetes was present. The risk of Covid-19 mortality in people with type 1 diabetes has been higher than with type 2 diabetes in some studies, but this has not been a consistent finding (Barron et al., 2020; Rawshani et al., 2021). However, an approximately ten-fold higher prevalence of type 2 versus type 1 diabetes ensures that the majority of deaths among people with diabetes occur in those with the type 2 form. Diabetes also increases the risk of other serious adverse Covid-19 outcomes, including development of severe respiratory distress, pneumonia and a need for treatment in the intensive care unit (ICU), assisted ventilation, or more drug therapies (Apicella et al., 2020; Seiglie et al., 2020; Zhu et al., 2020).

**FIGURE 2:**
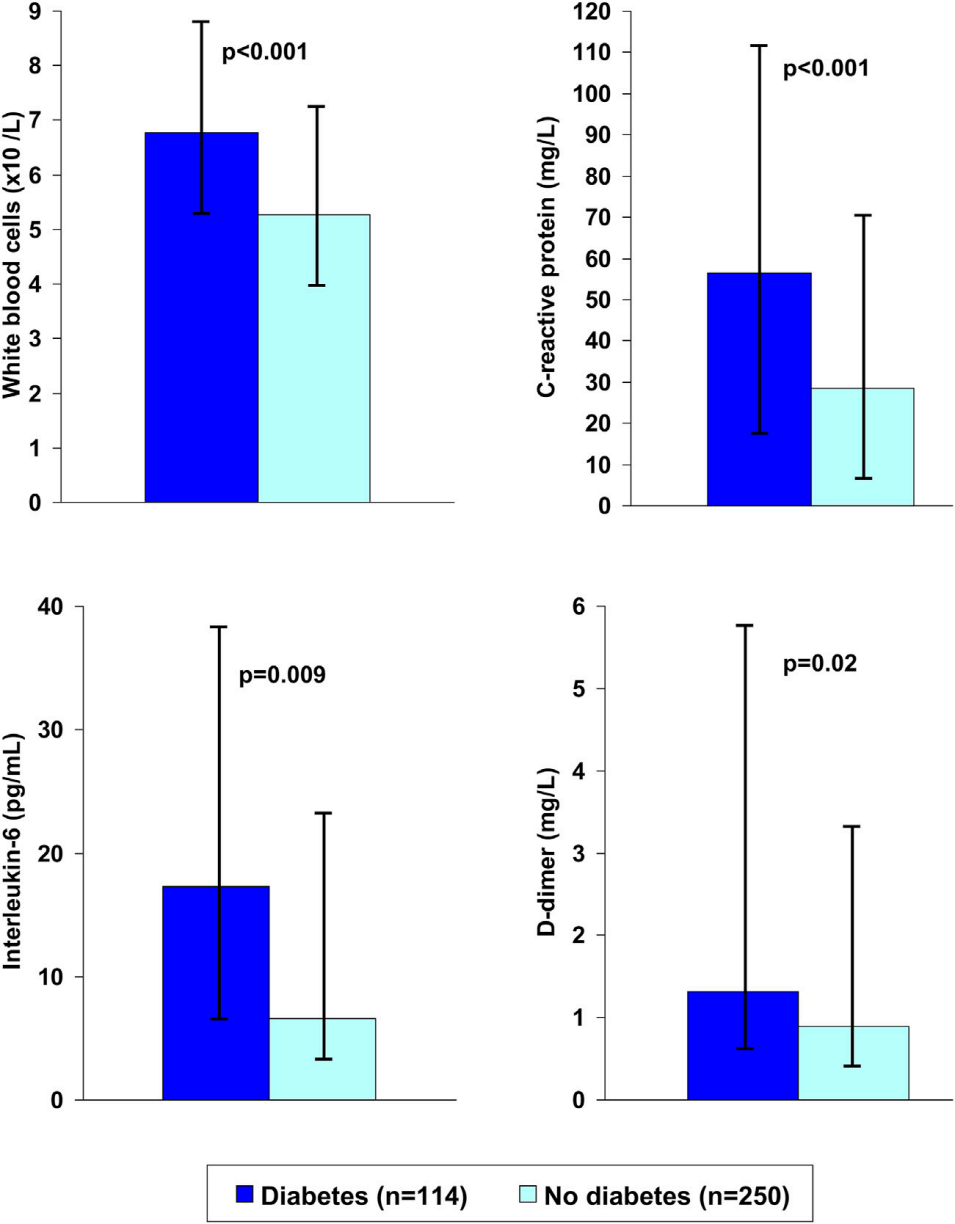
Markers of systemic inflammation and blood coagulability in patients with or without type 2 diabetes who died from Covid-19. Data are medians and interquartile ranges. Drawn from data presented by Xu et al., 2020.

Diabetes is associated with a range of cardiovascular risk factors, including overweight or obesity, systemic inflammation, insulin resistance, hypertension and a procoagulant state, as well as their clinical sequelae, notably coronary heart disease, heart failure and stroke (Dokken, 2008). Diabetes is also associated with an increased risk of chronic kidney disease and chronic obstructive pulmonary disease which are commonly encountered in patients with severe Covid-19 infection. Figure 2 shows the average levels of biomarkers of inflammation and hypercoagulability in patients with type 2 diabetes who either died from or survived Covid-19 (Xu et al., 2020). Many of these co-morbidities have themselves been associated with increased risk of serious adverse Covid-19 outcomes (Shi et al., 2020; Abu-Jamous et al., 2021), and the co-morbidities that commonly accompany diabetes are deemed to be at least partially additive to the metabolic disturbances of diabetes that aggravate the course of Covid-19 infection (Abdi et al., 2020; Ando et al., 2021; Diedisheim et al., 2021; Gregory et al., 2021; Rawshani et al., 2021). For example, the risks of ICU admission, need for ventilation and death increased as weight category increased (normal weight, overweight and obesity), and a diagnosis of diabetes conferred additional risk at each weight category (Seiglie et al., 2020). However, the presence of diabetes *per se* appears to impair the prognosis in people with Covid-19, in the absence of other comorbidities (Guo W. et al., 2020).

### Mechanisms for the Interaction of Covid-19 With Diabetes

Mechanisms through which the metabolic disturbances of diabetes increase the severity of Covid-19 infection have recently been reviewed in detail (Lim et al., 2021). The pro-inflammatory environment of obese and diabetic states facilitates an exaggerated cytokine response (cytokine storm) as noted with excessive plasma concentrations of C-reactive protein, interleukin-6, tumour necrosis factor-alpha, monocyte chemoattractant protein-1 and interferon-gamma in diabetes patients with severe Covid-19 infection (Gianchandani et al., 2020; Roberts et al., 2020; Zhu et al., 2020; Lim et al., 2021). Lymphocyte counts are notably reduced in diabetes patients with severe Covid-19 infection, particularly associated with greater hyperglycaemia (Zhang et al., 2020a; Gianchandani et al., 2020; Zhu et al., 2020; Zhang et al., 2021), and the effect of inflammatory cytokines to promote insulin resistance increases the hyperglycaemia in diabetes patients (Chen et al., 2015). Cellular damage by the viral infection, particularly inducing apoptosis, is aggravated by hyperglycaemia through increased reactive oxygen species, and there is evidence that the pro-coagulant state in diabetes promotes the thrombotic effects of severe Covid-19 infection (Feldman et al., 2020; Roberts et al., 2020; Singh et al., 2020; Lim et al., 2021). Severe Covid-19 infection is also prone to escalate inflammatory and fibrotic lesions, especially in lungs, heart, skeletal muscle and kidneys, which appear to confer lasting adverse effects (long-Covid) (Madjid et al., 2020; Raveendran and Misra, 2021). Expression of ACE2 by pancreatic beta-cells contributes to the susceptibility of these cells to Covid-19 attack, interrupting insulin secretory activity and accentuating hyperglycaemia (Feldman et al., 2020; Lim et al., 2021; Müller et al., 2021).

Whether possible alterations of ACE2 expression during antihypertensive treatment with ACE inhibitors and angiotensin receptor blockers could affect viral entry and transmission around the body remains unclear (Roberts et al., 2020). Overall, it is evident that diabetes and Covid-19 infection can create a vicious spiral in which the Covid-19 infection makes the diabetes worse and the diabetes makes the Covid-19 infection worse.

### Importance of Glycaemic Control

Blood glucose control seems to be important for the prognosis of Covid-19 infection: a study using propensity scoring to closely match two groups of 250 people with diabetes for cardiometabolic risk factors other than glycaemia (hypertension, cardiovascular disease, cerebrovascular disease, chronic kidney disease and diabetes duration) showed that good blood glucose control (3.9–10.0 mmol/L) vs. suboptimal blood glucose control decreased the risk of Covid-19 mortality by about 7-fold [HR 0.14 (95%CI 0.03, 0.60), *p* = 0.008] (Zhu et al., 2020). The well-controlled group also needed less treatment with antifungal agents, antibiotics, immunoglobulins, steroids, oxygen, and assisted ventilation. In addition, better glucose control has been associated with lower levels of markers of systemic inflammation (e.g., neutrophil count, IL-6 and C-reactive protein) and better immune function (higher lymphocyte count) (Huang Y. et al., 2020; Xu et al., 2020). In another study, people with diabetes and average blood glucose above versus below 7.8 mmol/L have been shown to require more treatment in the ICU and longer use of mechanical ventilation (Saand et al., 2021).

Registry studies have identified hyperglycaemia at admission as a significant, independent predictor of Covid-19 hospitalisation or mortality (O'Malley et al., 2021; Carrasco-Sánchez et al., 2021). The severity of hyperglycaemia appears to influence prognosis, appreciating that blood glucose can rise quickly during Covid-19 infection as seen with plasma glucose measurements but not with the gradual effect on HbA1c (Zhang Q. et al., 2020; Wang B. et al., 2021; Ling et al., 2021). Hyperglycaemia is associated with adverse Covid-19 outcomes whether or not patients have diabetes (Cai et al., 2020; Carrasco-Sánchez et al., 2021), with some suggestion of a more severe effect on Covid-19 prognosis for newly-diagnosed versus pre-existing diabetes, possibly associated with depressed respiratory function (Aggarwal et al., 2020; Fadini et al., 2020). For example, “secondary hyperglycaemia” (no diabetes history, fasting plasma glucose ≥7 mmol/L but HbA1c <6.5%) was associated with a doubling of the risk of a composite outcome of Covid-19 severity compared with pre-existing diabetes after hospitalisation for Covid-19 (Zhang et al., 2020b). Indeed, higher fasting glucose in Covid-19 patients without diabetes predicted increased circulating levels of inflammatory markers and reduced lymphocyte counts, which were in turn associated with a higher risk of mortality (Huang Y. et al., 2020).

### Impact of Covid-19 on Glycaemia

Covid-19 may exacerbate suboptimal blood glucose control in diabetes, as shown by a recent meta-analysis in which Covid-19 increased blood glucose by 2.2 mmol/L, on average (Chen J. et al., 2020). However, much greater effects on blood glucose are possible, as illustrated by a case report concerning a man with well-controlled metformin-treated type 2 diabetes (HbA1c 6.1%) who developed severe Covid-19 requiring ICU admission and assisted ventilation (Jornayvaz et al., 2020). His blood glucose rose to 15 mmol/L in the days following ICU admission, despite receiving 50 U/hour of rapid-acting insulin. This extreme manifestation of insulin resistance was unexplained, but may have been associated with severe systemic inflammation during a cytokine storm, which is recognised increasingly as an important component of the pathogenesis of life-threatening complications of Covid-19 (Chen and Quach, 2021).

## Metformin and Covid-19

### Metformin and Risk of Covid-19 Infection

A study of propensity score-matched cohorts of T2D patients receiving metformin or not receiving metformin in The Health Improvement Network (a United Kingdom primary care database, N = 10,183 for each cohort) reported similar hazard ratios [HR (95%CI)] between groups for development of suspected/confirmed Covid-19 [0.85 (0.67, 1.08)], or confirmed Covid-19 [0.80 (0.49, 1.30)] (Wang J. et al., 2021). A study from Korea (N = 11,892 people with T2D matched for use or non-use of metformin using propensity scoring) found a small, but significant, reduction in the risk of contracting Covid-19 in the metformin group [OR 0.88 (0.78, 0.99), *p* = 0.039] (Oh and Song, 2021). Overall, therefore, large database studies do not suggest a major influence of metformin on the risk of contracting Covid-19.

### Metformin and Severity of Covid Infection

Evaluations of the effects of metformin on clinical outcomes in people who develop Covid-19 have been of a retrospective design at this time, by necessity. Table 1 summarises findings from cohorts of outpatients who developed Covid-19 (some of whom were subsequently hospitalised for this condition) (Crouse et al., 2020; Do et al., 2020; Bramante et al., 2021a; Abu-Jamous et al., 2021; Wang J. et al., 2021; Ghany et al., 2021; Lally et al., 2021; Oh and Song, 2021) Several studies in outpatient populations demonstrated reduced risks of serious adverse outcomes such as hospitalisation or death due to Covid-19 in cohorts receiving versus not receiving metformin (Crouse et al., 2020; Do et al., 2020; Bramante et al., 2021a; Ghany et al., 2021; Lally et al., 2021). One of these studies demonstrated reduced mortality when metformin was prescribed *only after* testing positive for Covid-19 (Abu-Jamous et al., 2021). No significant influence of metformin treatment was seen in other studies (Do et al., 2020; Wang J. et al., 2021; Oh and Song, 2021).

**TABLE 1:** Overview of retrospective studies of the effect of metformin in people who became infected with SARS-Cov2 (Covid-19).

Ref	Design/patients	Summary of main findings in patients with Covid-19
Studies in outpatients		
Abu-Jamous et al. (2021)	456 inpatients with diabetes^a^ ^,^ ^b^	Reduced risk of death [0.19 (0.05, 0.70)] in diabetes patients who initiated “biguanide” (i.e. metformin) *only after* testing positive for Covid-19 (compared with patients who never received treatment)
Wang J. et al. (2021)	20,366 with T2D	No reduction in Covid-19 mortality in propensity score-matched cohorts of metformin users vs. non-users [HR: 0.87 (0.34, 2.20)]
Oh and Song, (2021)	11,892 with T2D	No effect of metformin on subsequent in-hospital Covid-19 mortality [OR 1.26 (0.81, 1.95), 0.301] between propensity-score matched cohorts of metformin users vs. non-users
Ghany et al. (2021)	1,139 US Medicare	Reduced RH for hospitalisation [0.71 (0.52, 0.86)], death [0.34 (0.19, 0.59)], ARDS [0.32 (0.22, 0.45)] for N = 392 on metformin vs. non-metformin, including patients without diabetes
Bramante et al. (2021a)	9,555 overweight subjects^c^	Metformin use (N = 676) vs. no metformin (N = 8,879) was associated with reduced mortality [OR 0.32 (0.15, 0.66), *p* = 0.002] and trends to reduced hospitalisation [OR 0.78 (0.58, 1.04) *p* = 0.087] and ICU admission [OR 0.68 (0.45, 1.02), *p* = 0.060]. Outcomes in 342 pairs of propensity score-matched metformin users and non-users were similar
Lally et al. (2021)	775 nursing home residents	Reduced risk of death [HR 0.48 (0.28, 0.84)] for metformin (N = 127) vs. no glucose-lowering drug (N = 476); no significant benefit for insulin or other glucose-lowering drug
Do et al. (2020)	1,865 with diabetes	No mortality difference between 469 metformin users and 95 non-users [HR 0.77 (0.44, 1.35), *p* = 0.052]. Mortality was higher for no metformin vs. no AD [HR 1.79 (1.04, 3.10), *p* = 0.036], but not for metformin vs. no glucose-lowering drug [HR 1.38 (0.97, 1.97), *p* = 0.076]
Crouse et al. (2020)	604 inpatients^a^	Metformin use vs. non-use among people with diabetes was associated with reduced mortality [OR 0.33 (0.13, 0.84), *p* = 0.0210] in people who tested positive for Covid-19 while in hospital for other reasons
Patients hospitalised for Covid-19		
(Li et al., 2020; Li et al., 2021)	131 with T2D	Overall, 85% recovered, 15% died. Reduced mortality vs. overall cohort in N = 37 receiving metformin (95% recovered/5% died, *p* = 0.02), N = 57 receiving acarbose (91% recovered/8% died, *p* = 0.04), or N = 13 who received both (all survived, p-0.03). No significant effect of other glucose-lowering drug (alone or in combination). Glucose-lowering drug use was maintained after hospitalisation for Covid-19^d^
Cheng et al. (2021)	407 with T2D	Reduced ICU admission for pre-admission metformin (N = 18) vs. no metformin (N = 32; 5.6 vs. 43.8%, *p* = 0.005). No significant difference between metformin and non-metformin groups in hospital. Non-significant trend to reduced mortality and need for invasive ventilation for metformin vs. non-metformin. No benefit for insulin vs. no insulin (trend towards adverse outcomes)
Bramante et al. (2021b)	6,255 with T2D or obesity	Reduced mortality with metformin in women [HR 0.785 (0.650, 0.951)] but not in men [HR 0.957 (0.82, 1.14), *p* = 0.689] or overall (HR 0.887 [0.782, 1.008]). Results from propensity score-matched cohorts were similar
Jiang et al. (2021)	328 with T2D	Reduced risk of ARDS for 100 metformin users vs. 228 non-users [OR 0.18 (0.05, 0.62), *p* = 0.007]; no significant effect on severity of Covid-19 or on 30-day mortality
Lalau et al. (2021)	2,449 with T2D	Reduced risk at 28 days post-admission of a composite of tracheal intubation or death [OR 0.783 (0.615, 0.996)] or death [OR 0.710 (0.537, 0.938)] for 1,496 metformin users vs. 953 non-users
Wargny et al. (2021)	2,796 with T2D	Metformin therapy was a significant predictor of earlier discharge (along with longer duration of symptoms): OR 1.40 (1.08, 1.81) for discharge within 28 d in the metformin vs. non-metformin group OR 1.40 (1.08, 181). Metformin was associated with reduced risk of death within 28 d 0.65 (0.45, 0.93)
Gao et al. (2020)	110 with T2D	More (*p* = 0.004) life-threatening complications in 56 metformin users (29%) vs. 54 non-metformin users (7.4%), with higher risk of disease progression during hospitalization [OR 3.964 (1.034, 15.194), *p* = 0.045]
Cheng et al. (2020)	1,213 with T2D	No effect of metformin (N = 678) vs. non-metformin (N = 535) on 30 day mortality [HR 1.65 (0.71, 3.86), *p* = 0.247]. Metformin was associated with less HF [HR 0.59 (0.41, 0.83), *p* = 0.003] but more acidosis [HR 2.73 (1.04, 7.13), *p* = 0.040], including lactic acidosis [HR 4.46 (1.11, 18.00), *p* = 0.036]; no difference for ARDS, DIC, AKI, acute heart injury
Luo et al. (2020)	283 with diabetes	Less (*p* = 0.01) in-hospital mortality for 104 metformin users (2.9%) vs. 179 non-users (12.3%); no difference for LOS.
Nafakhi et al. (2021)	192 with Covid-19 pneumonia	Metformin (and DPP4 inhibitor) associated with reduced overall LOS and LOS in the ICU.
Pérez-Belmonte et al. (2020)	2,666 with diabetes^e^	No association of metformin or other glucose-lowering drug with risk of mortality, composite ICU admission, mechanical ventilation, or in-hospital mortality, other complications in hospital, or LOS.
Chen Y. et al. (2020)	120 with diabetes^e^	No significant effect of metformin in in-hospital mortality, LOS, or assessment of poor prognosis

a Included in this part of the table because they were not hospitalised for Covid-19; 93% of the metformin group had T2D.

b From 1,253 positive results for SARS-CoV2 among 5,294 screened on admission to hospital for various causes.

c BMI >25 kg/m^2^.

d Before/after hospitalisation: metformin N = 37/37, acarbose N = 38/57.

e In the SEMI-COVID registry.AKI, acute kidney injury; ARDS, acute respiratory distress syndrome; DIC, disseminated intravascular coagulation; HF, heart failure; HR, hazard ratio; ICU, intensive care unit; LOS, length of (hospital) stay; OR, odds ratio; RH, relative hazard; T2D, type 2 diabetes; vs.: versus. Figures in square brackets are 95% Confidence Intervals.

The populations of the studies described all had type 2 diabetes, while other cohorts were selected for being overweight or obese (Bramante et al., 2021a), resident in a nursing home (Lally et al., 2021), an elderly US Medicare population (Ghany et al., 2021), or positive during screening for Covid-19 when hospitalised for other causes (Crouse et al., 2020), in each case with or without concomitant diabetes. Most metformin-treated patients with diabetes had type 2 diabetes as would be expected (84–99% of patients in studies where this information was provided (Bramante et al., 2021a; Ghany et al., 2021; Lally et al., 2021); thus, these studies involved comparison of a metformin group (mostly) with type 2 diabetes with a comparator group containing fewer patients with type 2 diabetes. Given that a diagnosis of diabetes is associated with a poorer prognosis in people with Covid-19 (see above), it is possible that a beneficial effect of treatment with metformin may have outweighed the potentially adverse influence of a higher prevalence of diabetes in the metformin versus non-metformin groups.

Table 1 also summarises the main findings of retrospective studies in patients identified at the time of admission to hospital for Covid-19 (Chen Y. et al., 2020; Cheng et al., 2020; Gao et al., 2020; Li et al., 2020; Luo et al., 2020; Pérez-Belmonte et al., 2020; Bramante et al., 2021b; Cheng et al., 2021; Jiang et al., 2021; Lalau et al., 2021; Li et al., 2021; Nafakhi et al., 2021; Wargny et al., 2021). Most of these studies demonstrated improved clinical outcomes in cohorts of patients who did versus did not receive metformin, including reductions in mortality, lesser need for highly intensive treatment [e.g., admission to the intensive care unit (ICU) or assisted ventilation], or reduced onset of acute respiratory distress syndrome (ARDS) (Cheng et al., 2020; Gao et al., 2020; Li et al., 2020; Luo et al., 2020; Bramante et al., 2021b; Cheng et al., 2021; Jiang et al., 2021; Lalau et al., 2021; Li et al., 2021; Wargny et al., 2021). One study reported that metformin reduced mortality only in women, with no significant effect in men, or in the overall population (Bramante et al., 2021b). There was more acidosis (including lactic acidosis) in metformin users than in non-users in another study, although mortality was unaffected and there was an apparent reduction in the frequency of heart failure in the metformin group (Cheng et al., 2020). The increased risk of acidosis could be due in part to accumulation of excess plasma metformin consequent to a rapid deterioration in renal function noted in more than 20% of patients with severe Covid-19 infection (Nadim et al., 2020). Acute renal failure is a contraindication that requires discontinuation of metformin as discussed later. A small study (113 subjects) reported an increase in life threatening complications of Covid-19 associated with metformin treatment during the hospital stay (Gao et al., 2020).

Systematic or pooled analyses that include studies listed in Table 1 provided similar results. A meta-analysis of 5 comparisons of metformin with a control group showed that metformin reduced mortality before [OR 0.45 (0.25, 0.81), *p* = 0.008] and after multivariable adjustment [OR 0.64 (0.43, 0.97), *p* = 0.035] in people with Covid-19 (Lukito et al., 2020). Another meta-analysis, reported as a letter to a journal, reported similar findings [OR for mortality 0.62 (0.43, 0.89) for metformin versus no metformin in this population] (Kow and Hasan, 2021). A systematic review concluded that metformin was associated with improved Covid-19 outcomes in 9/14 studies that recruited populations with or without diabetes (Zangiabadian et al., 2021).

Metformin is often used off-label in the management of prediabetes and polycystic ovary syndrome (Hostalek et al., 2015; Guan Y. et al., 2020; Hostalek and Campbell, 2021). Database analyses to specifically assess the effect of metformin on the severity of Covid-19 infections in these conditions have not been reported. However, it is possible that the actions of metformin that curb the severity of Covid-19 infection in type 2 diabetes might confer similar benefits to individuals receiving metformin for other conditions.

### Possible Mechanisms for Metformin on Severity of Covid Infection

**FIGURE 3:**
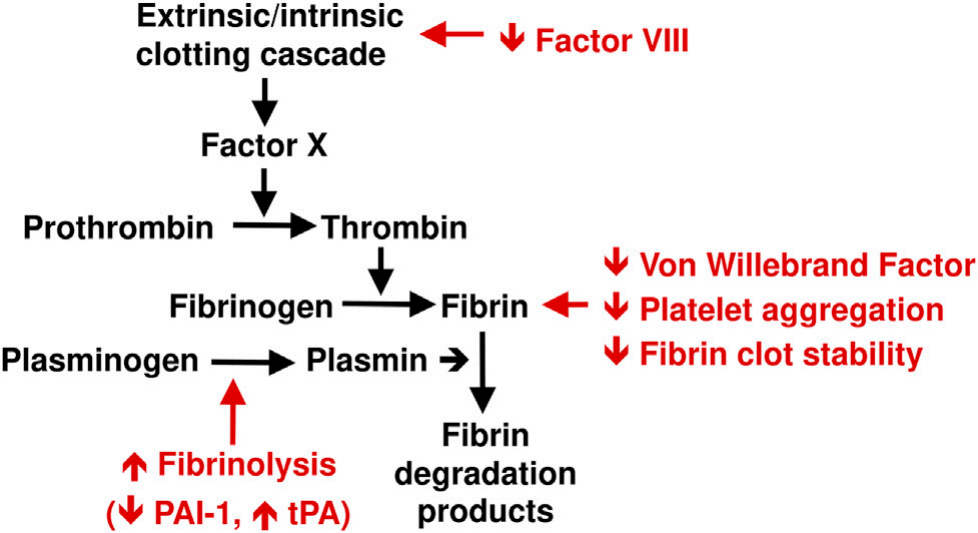
Schematic summary of antithrombotic effects of metformin (shown in red). See text for references. PAI-1, plasminogen activator inhibitor-1; tPA, tissue plasminogen activator.

Cardiovascular comorbidities increase the risk of severe, adverse Covid-19 outcomes, as described above. Metformin has been shown to reduce the risk of cardiovascular events and to increase survival in populations with type 2 diabetes in the randomised United Kingdom Prospective Diabetes Study (UKPDS) (UK Prospective Diabetes Study Group, 1998) and in large meta-analyses that included real world, observational data (Han et al., 2019; Zhang K. et al., 2020). Thus, reduced cardiometabolic comorbidities may explain, at least in part, the way in which metformin can improve the prognosis of people with Covid-19. A potential vascular benefit of metformin relates to the thrombotic effects of Covid-19 infection. Metformin is known to exert thrombolytic effects by reducing platelet aggregation, reducing the production of plasminogen-activator inhibitor-1 and altering fibrin structure as summarised in Figure 3 and described in detail elsewhere (Grant, 2003; Xin et al., 2016).

#### Metformin Anti-Inflammatory Effects

**FIGURE 4:**
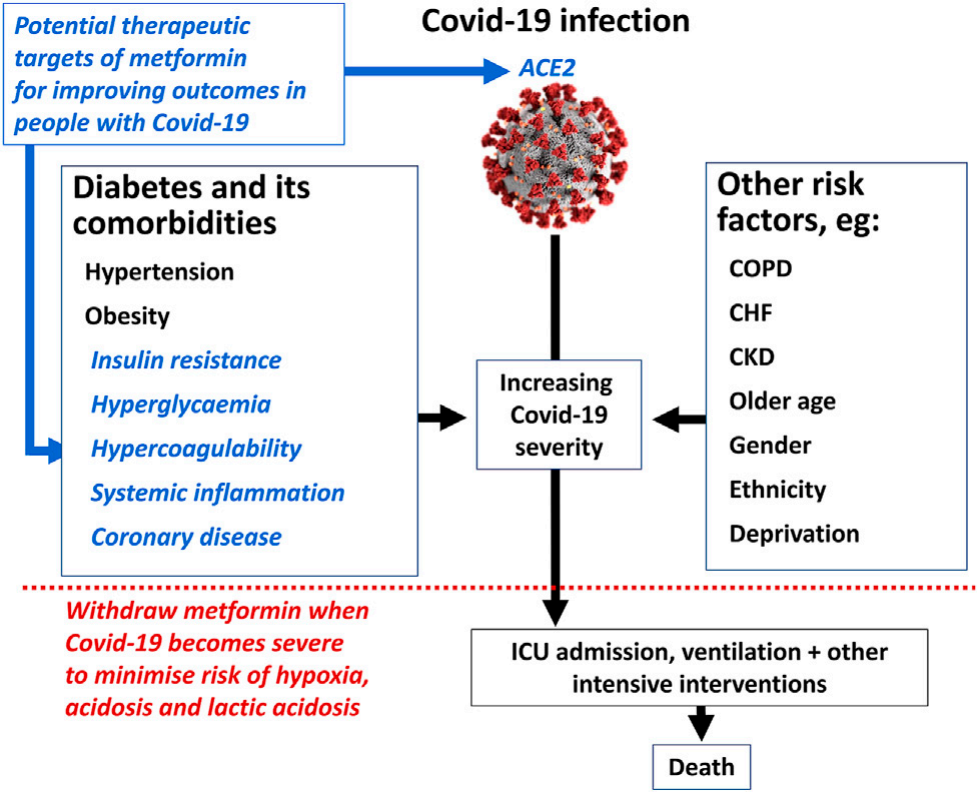
Summary of the potential mechanisms by which metformin might influence the course of severe Covid-19 infection. ICU, intensive care unit; CHF, congestive heart failure; CKD, chronic kidney disease; COPD, chronic obstructive pulmonary disease. See text for references. Image of coronavirus by permission of US Centers for Disease Control (https://www.cdc.gov/media/subtopic/images.htm).

The cellular mechanisms underlying metformin’s cardioprotective effect are likely to extend beyond its antihyperglycaemic effect (Campbell et al., 2017; Petrie et al., 2020). They include amelioration of several metabolic abnormalities, including insulin resistance (Du et al., 2013), hypercoagulability of the blood (Grant, 2003; Markowicz-Piasecka et al., 2020), and increased levels of markers of systemic inflammation (Saisho, 2015; Bai and Chen, 2021)that have been described in patients who progress to severe Covid-19 (Figure 2). (Zhu et al., 2020; Xu et al., 2020; Huang I. et al., 2020; Zhang Q. et al., 2020; Wargny et al., 2021; Govender et al., 2021; Wang et al., 2020) Accordingly, actions of metformin to reduce these risk factors may provide further explanation for at least some of the apparent benefit of metformin treatment in patients with Covid-19 (Figure 4). Indeed, lower circulating levels of inflammatory markers have been observed in metformin-treated patients with Covid-19, compared with non-metformin control groups (Chen Y. et al., 2020; Cheng et al., 2020; Al-Kuraishy et al., 2021).

**FIGURE 5:**
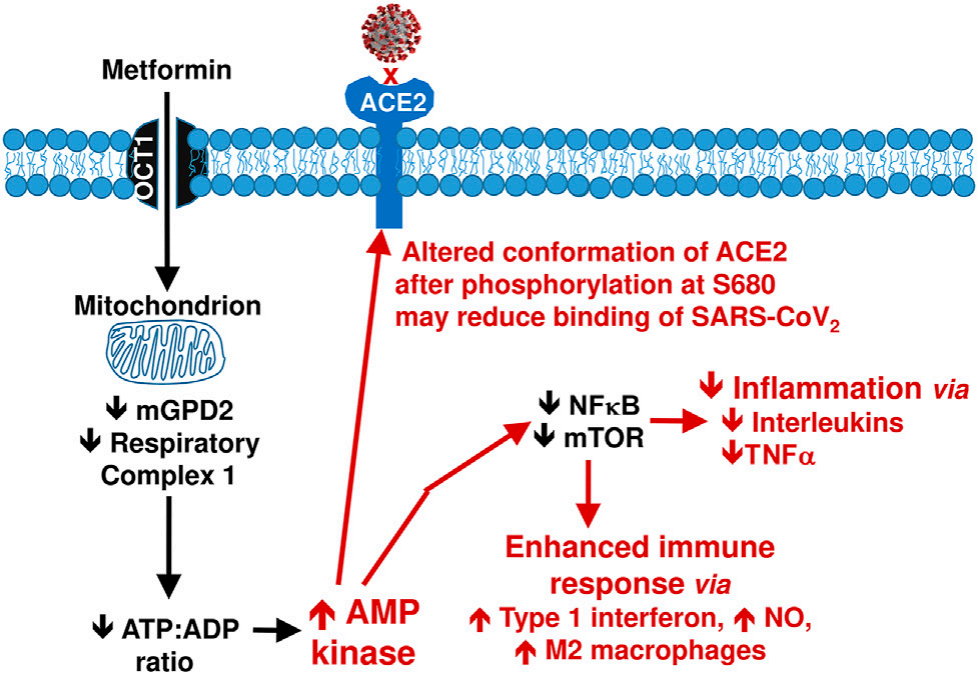
Summary of anti-inflammatory mechanisms of metformin arising *via* activation of the AMP kinase pathway. See text for references. NO, nitric oxide; TNF*α*, tumour necrosis factor alpha.

Inhibition of the mitochondrial respiratory chain at complex I by metformin increases the ratio of cellular ADP and AMP to ATP, which in turn activates the AMP-activated protein kinase (AMPK) pathway (Figure 5). (Vial et al., 2019) Metformin can also activate AMPK independently of adenine nucleotides (Hawley et al., 2002). Increased activity of AMPK will suppress signalling of the nuclear transcription factor, NFκB, mediated *via* downstream targets that include Forkhead box O (FoxO), peroxisome proliferator-activated receptor *γ* co-activator 1*α* (PGC-1*α*), tumour suppressor protein p53 and sirtuin-1 (Salminen et al., 2011). Suppression of NFκB signalling in turn reduces the production of inflammatory cytokines, notably interleukin-6 and TNF*α*, and this is deemed likely to explain much of the anti-inflammatory effect of metformin (Liu et al., 2017).

#### Metformin Anti-Viral Effects

Activation of AMPK by metformin also increases phosphorylation of the S680 serine residue of the ACE2 protein (Figure 4). (Zhang et al., 2018) ACE2 acts as a cell membrane enzyme and receptor which mediates endocytotic internalisation of the SARS-CoV2 virus by binding its spike protein. Phosphorylation of ACE2 is anticipated to alter the conformation of this molecule, which may reduce the binding of SARS-CoV2 virions at the cell membrane and thus impede access to the cell, representing a potential anti-Covid-19 mechanism of metformin (Malhotra et al., 2020). However, S680 phosphorylation of ACE2 may also slow the degradation of ACE2, potentially increasing its concentration. Plasma ACE2 promotes antihypertensive and anti-inflammatory effects *via* the production of angiotensin-(1–7) which then acts *via* the Mas receptor (Rodrigues Prestes et al., 2017; Rukavina Mikusic et al., 2021). This provides a further possible route through which metformin might reduce the pathology of Covid-19 infection (Vial et al., 2019). Figure 5 summarises potential mechanisms by which metformin may improve outcomes in people with Covid-19.

More speculatively, antiviral properties have been attributed to molecules containing a biguanide moiety, including metformin (Kathuria et al., 2021). Indeed, metformin was used in the management of influenza in 1950 (when it was known as “flumamine”), 7 years before its entry into clinical use for type 2 diabetes (Bailey et al., 2007). Further clinical research will be needed to define and quantify any potentially beneficial anti-viral effects of metformin specifically related to the course of infection with SARS-CoV_2_.

## Looking Ahead: Should Metformin Be Discontinued With Covid-19 Infection

Most studies described above reported either no effect, or a potential benefit, on clinical outcomes associated with pre-existing metformin treatment after contracting Covid-19. These data are all retrospective, however, which limits their utility for guiding future practice. Also, important information is lacking at this time, for example relating to exactly when, and for whom, metformin may have been withdrawn during hospitalisation. In addition, only observational data on the effects of metformin in people with Covid-19 are available at present, and such data need to be interpreted with caution, due to risk of confounding. Appropriately designed clinical trials are needed to explore the reasons for the apparent amelioration of the course of Covid-19 infection in metformin treated type 2 diabetes subjects in the retrospective studies described herein.

The two studies that suggest potential harms of metformin treatment on outcomes in patients hospitalised for Covid-19 (Cheng et al., 2020; Gao et al., 2020) are of particular interest in evaluating the safety of metformin for people with type 2 diabetes who develop Covid-19. In one study in people with type 2 diabetes, the prevalence of life-threatening complications of Covid-19 was rare at admission (1.8% in the metformin group, and 0% in the control group) (Gao et al., 2020). However, during the stay in hospital 29% of the metformin group and only 7% of the control group required ICU admission (*p* = 0.004), with a 4-fold increase in the risk of life-threatening complications in the metformin vs. non-metformin groups (Gao et al., 2020). The second study demonstrated an association of metformin treatment with a 2.5-fold increased risk of acidosis (*p* = 0.032) and a 4.7-fold increase in lactic acidosis (*p* = 0.010) during hospitalisation for Covid-19 (Cheng et al., 2020). Moreover, these risks were much higher where renal function was compromised (eGFR <60 ml/min/1.73 m^2^; risk increases 5.2- and 3.9-fold for acidosis and lactic acidosis, respectively), or when metformin was given at a high dosage (2–3 g/day; risk increases 12.8- and 22.6-fold for acidosis and lactic acidosis, respectively). The acute decline in renal function that affects >20% of hospitalized patients with Covid-19 infection and >50% of patients admitted to ICU (Nadim et al., 2020) is therefore a particular caution for continued use of metformin. It is generally recommended that the dose of metformin should not exceed 1,000 mg/day if GFR declines to below 45 ml/min/1.73 m^2^, and metformin should be discontinued if the GFR declines to below 30 ml/min/1.73 m^2^. Accumulation of excess metformin that occurs when drug elimination is impeded by renal impairment is likely to make an important contribution to the risk of acidosis, and especially lactic acidosis due to increased metformin-associated anaerobic metabolism. Anaerobic metabolism will also aggravate the hypoxemia of acute respiratory distress and this represents another caution for discontinuation of metformin.

Acute metabolic acidosis, conditions with the potential to impair renal function (including severe infection) or tissue hypoxia (including respiratory failure or severe congestive heart failure) are all contra-indications for treatment with metformin, which were put in place to minimise the risk of lactic acidosis with this treatment (DeFronzo et al., 2016). Nevertheless, metformin treatment appears to be generally safe for most people in the early stages of Covid-19 infection, according to the data summarised above. However, withdrawal of metformin once Covid-19 infection becomes severe represents a reasonable and pragmatic precaution for metformin administration (Figure 4). Such an approach may reduce the potential for developing either life-threatening complications of Covid-19, or lactic acidosis events associated with metformin.

## Conclusion

People with diabetes are at a markedly increased risk of adverse outcomes related to Covid-19 infection. Retrospective data suggest that type 2 diabetes patients receiving treatment with metformin at the time of hospitalisation with Covid-19 infection appear to have better outcomes in terms of reduced need for intensive care and reduced mortality compared with patients not receiving metformin when admitted. While the literature does not distinguish between patients who continued or discontinued metformin during hospitalisation, evidence supports the use of metformin when Covid-19 infection is not severe. Due to the risk of hypoxaemia, acute renal disease, cardiovascular complications and acidosis, it is appropriate to consider withdrawing metformin when Covid-19 becomes more severe. Retrospective studies are by nature hypothesis generating, and there is a need for prospective study of the effects of metformin in people with Covid -19.

## Take Home Message

Metformin may be continued for most people with type 2 diabetes who present with milder forms of Covid-19, but should be withdrawn when Covid-19 becomes more severe to reduce the potential for aggravation of the risk of acidosis and hypoxaemia.
